# Do Plant-Bound Masked Mycotoxins Contribute to Toxicity?

**DOI:** 10.3390/toxins9030085

**Published:** 2017-02-28

**Authors:** Silvia W. Gratz

**Affiliations:** Rowett Institute of Nutrition and Health, University of Aberdeen, Foresterhill, Aberdeen AB25 2ZD, UK; s.gratz@abdn.ac.uk; Tel.: +44-1224-438-675

**Keywords:** masked mycotoxins, deoxynivalenol, trichothecenes, zearalenone, absorption, hydrolysis, intestine

## Abstract

Masked mycotoxins are plant metabolites of mycotoxins which co-contaminate common cereal crops. Since their discovery, the question has arisen if they contribute to toxicity either directly or indirectly through the release of the parent mycotoxins. Research in this field is rapidly emerging and the aim of this review is to summarize the latest knowledge on the fate of masked mycotoxins upon ingestion. *Fusarium* mycotoxins are the most prevalent masked mycotoxins and evidence is mounting that DON3Glc and possibly other masked trichothecenes are stable in conditions prevailing in the upper gut and are not absorbed intact. DON3Glc is also not toxic per se, but is hydrolyzed by colonic microbes and further metabolized to DOM-1 in some individuals. Masked zearalenone is rather more bio-reactive with some evidence on gastric and small intestinal hydrolysis as well as hydrolysis by intestinal epithelium and components of blood. Microbial hydrolysis of ZEN14Glc is almost instantaneous and further metabolism also occurs. Identification of zearalenone metabolites and their fate in the colon are still missing as is further clarification on whether or not masked zearalenone is hydrolyzed by mammalian cells. New masked mycotoxins continuously emerge and it is crucial that we gain detailed understanding of their individual metabolic fate in the body before we can assess synergistic effects and extrapolate the additive risk of all mycotoxins present in food.

## 1. Introduction

**Figure 1:**
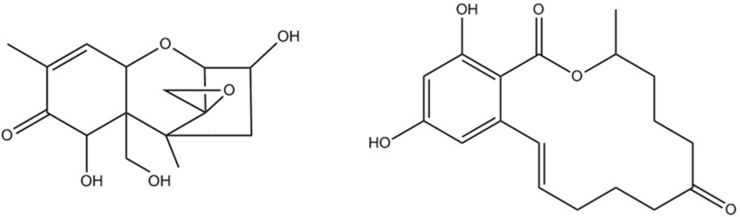
Chemical structures of deoxynivalenol (**left**) and zearalenone (**right**).

Mold infestation is an intractable problem in agricultural production affecting especially cereal crops, nuts, and fruits either during plant growth or post-harvest. The fungal genera *Aspergillus*, *Pencillium*, and *Fusarium* are most critical with regards to mycotoxin production. Mycotoxins are secondary fungal metabolites which are highly toxic and their stability and persistence in the food network are of major concern. The most important groups of mycotoxins include aflatoxins and *Fusarium* mycotoxins [1]. Aflatoxins are class 1 human carcinogens with a global prevalence of 18% in a survey of over 18,000 samples [2], but are not subject to plant metabolism and are therefore not further discussed in this review. Among the *Fusarium* mycotoxins, the three major groups of toxins are trichothecenes, zearalenone compounds, and fumonisins. The most common trichothecene, deoxynivalenol, was found in 73% of tested samples whereas zearalenone occurred in 56% of all samples globally [2]. Trichothecenes have a sesquiterpenoid basic structure with variable side chains and an epoxide group which is linked to toxicity (Figure 1). While type A trichothecenes, namely T2-toxin, HT2-toxin, and diacetoxyscirpenol (T2, HT2, DAS), are more potent toxins (tolerable daily intake TDI for T2 + HT2 is 0.1 µg/kg body weight [3]), it is the type B trichothecenes, especially deoxynivalenol (DON, TDI 1 µg/kg body weight [4]) and nivalenol (NIV), that are most commonly detected in cereals [5]. Trichothecenes compromise small intestinal nutrient absorption and epithelial barrier function and are immunotoxic [6,7,8]. Zearalenone (ZEN) and its hydroxylated derivatives α- and β-zearalenol (α-ZEL, β-ZEL) are lactone derivatives of phenolic resorcyclic acid which cause strong estrogenic effects and reproductive toxicity (TDI 0.25 µg/kg body weight) [1,5]. The third group of *Fusarium* mycotoxins are fumonisins, which are carcinogenic mycotoxins mainly found in corn products [9], but they are not further discussed here as to date no plant-derived masked forms have been described.

## 2. Masked Mycotoxins

In addition to the well characterized fungal mycotoxins, plant-derived mycotoxin metabolites have emerged as important co-contaminants in cereals [10]. These plant-derived, covalently bound mycotoxin conjugates are also termed masked mycotoxins. *Fusarium* mycotoxins (especially DON, ZEN, T2, HT2, and NIV) are the most prominent targets for plant metabolism utilizing phase II conjugation reactions with small molecules such as monosaccharides, glutathione, or sulfates [11]. The most commonly detected masked mycotoxin conjugates are β-linked glucose-conjugates of trichothecenes (DON3Glc, NIV3Glc, HT2Glc) and zearalenone (ZEN14Glc) [11,12] whereas both α- and β-glucoside have been reported for T2-toxin [13]. Masked mycotoxins have been detected in various cereals and cereal based foods. DON3Glc is found in wheat, maize, oats, and barley at relative proportions of 20%–70% of free DON [10]. NIV3Glc has been reported to occur in wheat at 12%–27% of NIV [14] and T2Glc and HT2Glc have been found in wheat and oats [15]. ZEN14Glc as well as α- and β-ZEL14Glc have been reported in breads and breakfast cereals at relative proportions of 20%–100% of free ZEN [16] and ZEN16Glc has recently been identified as a novel masked ZEN metabolite [17]. In addition to these single-sugar conjugates, di-, tri-, and tetra-glucosides as well as mixed disaccharides and malonyl-glucosides have been described for DON, T2, HT2, and ZEN [18,19]. Glutathione-conjugates of DON have been identified in barley [20], wheat, and oats [21]. Sulfo-conjugates have been described for ZEN (ZEN14S) in naturally contaminated animal feed [22] and for DON (DON3S, DON15S) in artificially contaminated wheat [23,24]. Despite this growing list of masked mycotoxins, the literature assessing their fate in the gut and predicting their contribution to toxicity is rather limited. Mainly hampered by the limited availability of purified compounds, most work has been performed on DON3Glc and ZEN14Glc with some studies investigating other masked mycotoxins (summarized in Table 1). No work has been performed to date on intestinal hydrolysis, absorption, or toxicity of more complex mycotoxin-oligosaccharide conjugates.

## 3. Toxicity of Masked Mycotoxins

Masked mycotoxins have long been thought to have limited bioavailability in the gut due to their covalent binding, and hence do not contribute to toxicity. In silico analysis predicted that DON3Glc cannot sterically bind to the ribosomal 60S subunit A-site pocket, the target for DON-induced ribosomal toxicity, which was confirmed in vitro as a lack of downstream activation of MAPK in Caco-2 cells [30]. As a result, DON3Glc (0–10 µM) was found non-toxic to Caco-2 cells as it did not decrease cell viability or disrupt intestinal barrier integrity [30] and was significantly less cytotoxic than free DON and acetylated DON when using flow cytometry to determine cell viability, apoptosis, and necrosis in IPEC-J2 porcine intestinal cells [31]. DON3Glc is also less immunotoxic than free DON [30,37]. Ex vivo DON3Glc did not up-regulate pro-inflammatory cytokine gene expression or induce functional and histological changes in porcine intestinal tissue explants [30]. In vivo DON3Glc was also shown to be largely incapable of eliciting pro-inflammatory cytokine and chemokine mRNA responses observed by DON in mice 2 and 6 h after oral exposure to 2.5 mg/kg body weight [37]. At similar dose levels (2.5–10 mg/kg body weight), DON3Glc was also found to be less potent at stimulating plasma elevation of gut satiety peptides and anorectic responses in mice and was markedly less potent at inducing emesis in minks compared to parent DON [38]. Overall, toxicity studies in various models focusing on different modes of toxicity all agree that the toxic potency of DON3Glc is much lower compared to free DON.

Toxicity studies of other masked mycotoxins are scarce. MethylthioDON, a product of the glutathione pathway in plants, was found to be 11-fold less toxic than DON in an in vitro translation assay testing the inhibition of protein biosynthesis [39]. DON3S was found to be non-toxic in the same in vitro translation assay [23,24], although a proliferative effect was reported in HT-29 colon cancer cells in vitro, which will need further investigation [24].

ZEN14Glc has been tested in vitro and found to be non-cytotoxic to MCF-7 human breast cancer cells (1 µM, 6 h) and Caco-2 cells (20 and 40 µM, 6 h) [41,42], and to exhibit reduced estrogen receptor binding capacity compared to ZEN [45]. ZEN16Glc is also non-cytotoxic in Caco-2 cells [41]. ZEN14S is also non-estrogenic in MCF-7 cells [43] and 40% less potent than ZEN in a rat uterus enlargement bioassay in vivo [44].

## 4. Digestion and Absorption Masked Mycotoxins in the Upper Gastro-Intestinal (GI) Tract

### 4.1. In Vitro Digestion

Several in vitro studies have so far confirmed that DON3Glc is resistant to digestive juices of the upper GI tract (saliva, stomach, pancreas, bile) without any significant release of DON [25,26,27,28]. No such studies have been performed with DON3S or DON-GSH. Similarly, other important masked trichothecenes (T2αGlc, T2βGlc, HT2Glc, and NIV3Glc [13,28]) and masked zearalenone compounds (ZEN14Glc, ZEN14S, αZEL14Glc, βZEL14Glc [26,28]) have been shown in vitro to be unaffected by conditions prevailing in the upper GI tract. Masked trichothecenes (DON3Glc, NIV3Glc, T2αGlc at 2–2.3 nmol/mL) have also been shown not to be transported across intestinal epithelial monolayers (Caco-2 or Caco-2/TC7) [27,28] while their free parent compounds were transferred to various degrees (38% of DON, 6% of NIV, 42% of T2/HT2 transported after 24 h).

Results for masked ZEN14Glc are less in agreement. Our recent study has shown that neither ZEN14Glc nor α- and β-ZEL14Glc were transported across the intestinal epithelial monolayers with 91%–93% of a 2 µM dose being recovered from the apical cell culture medium without any significant release of free ZEN after 24 h of exposure [28]. In contrast to this, ZEN14Glc has been shown to be hydrolyzed by Caco-2 cells with 20% of a 40 µM dose of apical ZEN14Glc being released as apical ZEN after 4 h of incubation, but no hydrolysis of ZEN16Glc was reported [41]. Whether this discrepancy stems from differences in dosing levels, Caco-2 cell clones, and/or culture conditions (e.g., DMEM versus RPMI culture medium) remains to be elucidated in future studies. Another in vitro study showed the hydrolysis of ZEN14Glc to ZEN and further metabolism by the human breast cancer cell line MCF-7, but the authors used complete culture medium supplemented with fetal bovine serum and suggest that serum components rather than cells may have hydrolyzed the masked compound [42]. Subsequently the hydrolysis of ZEN14Glc has been confirmed in bovine whole blood, plasma, and serum [46]. More work is required to clarify the issue of potential uptake and hydrolysis of ZEN14Glc by mammalian cells.

### 4.2. In Vivo Digestion and Absorption

The first report on masked mycotoxins in vivo dates back to 1990 when Gareis et al. [47] observed in one pig that ZEN14Glc (600 µg/d over 14 days) was decomposed during digestion and that ZEN and ZEL were detectable urinary and fecal metabolites. Authors report complete hydrolysis of ZEN14Glc but no associated clinical signs of estrogenic activity. Over two decades later the next in vivo study was performed as short term dosing (55 min) in two rats [32]. ZEN14Glc was rather unstable in the stomach (only 35% and 46% of dose recovered as ZEN14Glc) and rapidly hydrolyzed to free ZEN (16% and 19%). Only minimal amounts of intact ZEN14Glc were detectable in the intestine (0.5% in the small intestine, 2.5% in the colon). From these two studies, it appears that ZEN14Glc is rapidly hydrolyzed in vivo and therefore very likely to contribute to toxicity, but further work is needed to fully understand toxicokinetics and species differences.

Amongst trichothecenes, most in vivo studies have been performed with DON3Glc comparing its metabolism, absorption, and excretion to DON. In the rat study by Versilovskis [32], DON3Glc (25 µg/d or approximately 90 µg/kg body weight) was mainly recovered intact in the stomach (37% and 51% of the dose) whereas the release of free DON was negligible (2% in the stomach). In the small and large intestine, only traces of DON3Glc (1%–3%) and no free DON were found. Another single dose study in rats (2.0 and 3.1 mg/kg body weight of DON and DON3Glc, respectively) found fecal excretion as the major route of toxin elimination after DON and DON3Glc dosing (approximately 65% of an oral dose for both toxins over 48 h [33,34]). Sulfonate metabolites of DON and DOM-1 were major fecal metabolites for rats dosed with DON and DON3Glc, whereas DON3Glc-sulfonate was only found after dosing with DON3Glc. Fecal free DON plus DOM-1 comprised about 15% of the total dose of both toxins. Urinary metabolites only comprised 10% of the dose for DON and 5% of the dose for DON3Glc, suggesting low bioavailability of DON in rats, and even lower bioavailability of DON3Glc. The fate of DON3Glc in the pig appears to be rather different. Following oral dosing of DON3Glc (116 µg/kg body weight), urinary DON was the main excretion product after 24 h (21.6% of the dose) followed by urinary DON-glucuronides (10.2%), DOM-1 (5.9%), and DON3Glc (2.6%), as well as fecal DOM-1 (1.8%), adding up to a total recovery of 42.1% of the dose [35]. The recovery of an equimolar dose of free DON was twice as high (84.8% as urinary DON and DON-glucuronides) in the same study. Possible explanations for the low recovery of DON3Glc were the formation of DOM-1 glucuronides (which were not detected in that study) and incomplete elimination of all metabolites within 24 h as a result of the delayed large intestinal breakdown of DON3Glc. Another toxicokinetic study in pigs comparing oral dosing of DON and DON3Glc (36 and 55.7 µg/kg body weight, respectively over 8 h) found that DON3Glc is absorbed (as DON) much more slowly than DON (time to maximum plasma concentration is 225 min for DON3Glc versus 44 min for DON) and much less efficiently (absorbed fraction 16.1% for DON3Glc versus 81.3% for DON). DON3Glc was not absorbed intact at all (absolute oral bioavailability 0%). In broilers, neither mycotoxin is absorbed efficiently (5.6% and 3.8% oral bioavailability for DON and DON3Glc, respectively), possibly explaining this species’ low susceptibility to DON [36]. In summary, oral bioavailability and absorption of DON3Glc in pigs is significantly lower and slower compared to DON. Nevertheless, urinary excretion is the main route of toxin elimination suggesting hydrolysis of DON3Glc in the lower gut and significant uptake of DON metabolites from the colon. A more complete picture of all possible metabolites monitored over a longer period (48–72 h) is still lacking. Other species (rat and broiler chicken) do not appear to absorb DON3Glc or DON very efficiently, which may explain their lower susceptibility to DON compared to pigs. However, when predicting human exposure, the pig is thought to be the best model, raising concern about the fate of DON3Glc in the human large intestine.

As far as the other masked mycotoxins are concerned, there is very limited evidence on the hydrolysis of ZEN14Glc in vivo and no evidence on any other masked compounds. This gap of knowledge clearly needs to be addressed in future work.

## 5. Degradation of Masked Mycotoxins in the Lower GI Tract

The contribution of intestinal microbiota to the breakdown and metabolism of dietary compounds and xenobiotics is being widely recognized, but microbiome activity is difficult to study and predict in vivo. Hence microbial hydrolysis of masked mycotoxins has been studied in vitro using human fecal microbiota or single isolates of human gut microbes. Several studies have demonstrated complete microbial hydrolysis of DON3Glc after 24 h of incubation [25,26,28,29]. Kinetics of hydrolysis are similar for DON3Glc and NIV3Glc, but slower for T2αGlc in mixed fecal incubations [28]. Microbial hydrolysis rates of T2αGlc and T2βGlc were found to be similar, but T2βGlc was cleaved to T2 and HT2 while 30% of T2αGlc was converted to unknown metabolites, which could include HT2Glc, T2-triol, or T2-tetrol [13]. The identity and toxicity of these metabolites remains to be elucidated. Kinetic studies with purified recombinant glycosyl hydrolase (GH3) enzymes derived from *Lactobacillus brevis* and *Bifidobacterium adolescentis* showed significantly slower kinetics for the hydrolysis of NIV3Glc compared to DON3Glc, although assays were performed at 10-fold lower concentrations [40]. Regarding masked ZEN compounds, ZEN14Glc, ZEN14S, as well as α- and β-ZEL14Glc are all very rapidly hydrolyzed by mixed fecal microbiota with recovery of intact masked compounds dropping below 20%–40% after 30 min of incubation [26,28]. Furthermore, the microbial metabolism of ZEN14Glc leads to complete disappearance of known metabolites (ZEN14Glc, ZEN, α- and β-ZEL), suggesting further transformation to yet unidentified metabolites.

The covalent binding of masked mycotoxins to sugar residues significantly limits their absorption in the small intestine and acts as a delivery mechanism for masked mycotoxins to the colon. In the colon, simple masked mycotoxins have been shown to be efficiently hydrolyzed and free mycotoxins released. It is highly likely that di-, tri-, and tetra-saccharide masked mycotoxins can also be cleaved by the gut microbiome, as the metabolic network of gut microbiota comprises a vast arsenal of carbohydrate-active enzymes capable of degrading a multitude of polysaccharides and glycans [48]. It remains unknown how early that mycotoxin release will occur in the large intestine and how efficient the colonic absorption may be.

In addition to releasing free parent mycotoxins, further microbial metabolism has also been reported for DON [29]. DOM-1 can be used as a purely microbe-derived mycotoxin metabolite to extrapolate colonic uptake. In pigs dosed with DON, no DOM-1 was detectable in feces or urine whereas dosing with DON3Glc resulted in detectable levels of DOM-1 in feces and urine (2.1% and 5.9% of dose, respectively) [45]. DON3Glc exposure in pig therefore results in a urinary DOM-1 ratio of 18% of urinary DON (free + conjugated) whereas DON exposure results in 0% DOM-1. In humans, we have observed urinary DOM-1 ratios at 1.3%–7.7% of urinary DON (free + conjugated) in individuals harboring a microbiota capable of DOM-1 production [46]. These individuals will most likely have been co-exposed to DON and DON3Glc through their habitual diet. Dietary DON would then be mainly absorbed in the small intestine and excreted as urinary DON whereas dietary DON3Glc would be delivered to the colon, hydrolyzed, and partially metabolized to DOM-1 by microbiota and would thereafter be absorbed and excreted as urinary DON and DOM-1. However, conclusive evidence on colonic absorption of microbial mycotoxin metabolites and their potential role in low-grade colonic toxicity needs to be generated through future investigations. It will be crucial that future toxicokinetic studies and risk assessments take into account the contributions of microbial release and metabolism of mycotoxins and other xenobiotics, as well as their colonic absorption to gain a complete understanding of the fate of these compounds in the body [46].

## 6. Summary and Conclusions

The literature indicates that masked mycotoxins are significantly less toxic than their free parent compounds, although only DON3Glc has been studied thoroughly. Masked mycotoxins are also stable under conditions present in the upper GI tract and their intestinal absorption appears significantly lower than the free mycotoxins. However, the difference between the two most studied masked mycotoxins, DON3Glc and ZEN14Glc, is striking. DON3Glc is rather inert, withstands small intestinal digestion, and is not absorbed into epithelial cells or systemic circulation, but is efficiently hydrolyzed into free DON (and occasionally DOM-1) by gut microbiota. DON itself is also stable, circulating through the body and being excreted mainly as glucuronidated DON via urine. ZEN14Glc on the other hand appears much more biologically reactive with hydrolysis to free ZEN, hydroxylation to α- and β-ZEL, and further metabolism to yet undetermined metabolites occurring in blood, cell culture medium (when supplemented with fetal serum), and possibly epithelial cells. Upon contact with gut microbiota, ZEN14Glc is hydrolyzed and metabolized almost instantaneously into yet unknown metabolites. The fate of the resulting metabolites in terms of colonic absorption and toxicity remains to be determined. It is also unknown how steriochemically different isomers (e.g., ZEN16Glc) or more complex di- and trisaccharide masked compounds may be hydrolyzed by microbiota.

In conclusion, masked mycotoxins may not be viewed as a homogenous group of contaminants but rather as a complex mixture of different plant metabolites of various classes of mycotoxins, and their entirety has been termed as ‘maskedome’ [49]. It is crucial that we gain a detailed understanding of the fate of each individual masked mycotoxin before we allow the generalization of results and prediction of the metabolic fate of novel masked compounds.

## Figures and Tables

**Table 1:** Summary of published studies investigating digestion, absorption and toxicity of masked mycotoxins.

Mycotoxin	Study Design	Stomach	Small Intestine	Large Intestine	Absorption	Toxicity
**DON3Glc**	In vitro	Stable [25,26,27,28]	Stable [25,26,27,28]	Hydrolysed [25,26,28,29]	<1% [27,28]	<DON [30,31]
	In vivo	Stable [32]	Stable [32]	Hydrolysed (indirect evidence) [32,33,34,35]	<DON [32,33,34,35,36]	<DON [37,38]
**DON3S**	In vitro	?	?	?	?	<DON (translation assay) [23,24] but proliferative (HT-29 cells) [24]
**DON-GSH**	In vitro	?	?	?	?	<DON (translation assay) [39]
**T2αGlc**	In vitro	Stable [13,28]	Stable [13,28]	Hydrolysed [13,28]	0% [28]	?
**T2βGlc**	In vitro	Stable [13]	Stable [13]	Hydrolysed [13]	?	?
	In vivo	?	?	?	?	?
**HT2Glc**	In vitro	?	?	Hydrolysed [40]	?	?
	In vivo	?	?	?	?	?
**NIV3Glc**	In vitro	Stable [28]	Stable [28]	Hydrolysed [28,40]	0% [28]	?
	In vivo	?	?	?	?	?
**ZEN14Glc**	In vitro	Stable [26,28]	Stable [26,28]	Hydrolysed and metabolised [26,28]	<1% [31], 2.5% [41] >20% Hydrolysis [41]	Non-cytotoxic (Caco-2, MCF-7 cells) [41,42]
	In vivo	18% release [32]	Not recovered [32]	Not recovered [32]	?	?
**ZEN16Glc**	In vitro	?	?	?	<1% [41]	Non-cytotoxic (Caco-2 cells) [41]
	In vivo	?	?	?	?	?
**ZEN14S**	In vitro	Stable [26]	Stable [26]	Hydrolysed and metabolised [26]	?	<ZEN [43]
	In vivo	?	?	?	?	40% < ZEN [44]
**αZEL14Glc**	In vitro	Stable [28]	Stable [28]	Stable [28]	<1% [28]	<ZEN [45]
	In vivo	?	?	?	?	?
**βZEL14Glc**	In vitro	Stable [28]	Stable [28]	Stable [28]	0% [28]	?
	In vivo	?	?	?	?	?

?: No studies published.
